# Supplementary material for: Role and mechanism of NCAPD3 in promoting malignant behaviors in gastric cancer
Source: Front Pharmacol. 2024 Apr 22;15:1341039. doi: 10.3389/fphar.2024.1341039 (PMC11070777; doi:10.3389/fphar.2024.1341039)
Supplement: Supplementary file 11 [file DataSheet2.ZIP › GSEA/GO_CC/my_analysis.Gsea.1599461408869/GO_CATALYTIC_COMPLEX.html]

Details for gene set GO\_CATALYTIC\_COMPLEX[GSEA]

|  || Dataset | filtered\_dataset.sample\_info.cls#WT\_versus\_NCAPD3\_MUT |
| Phenotype | sample\_info.cls#WT\_versus\_NCAPD3\_MUT |
| Upregulated in class | WT |
| GeneSet | GO\_CATALYTIC\_COMPLEX |
| Enrichment Score (ES) | 0.18636574 |
| Normalized Enrichment Score (NES) | 1.3562889 |
| Nominal p-value | 0.08677098 |
| FDR q-value | 0.56017566 |
| FWER p-Value | 1.0 |
Table: GSEA Results Summary

  

Fig 1: Enrichment plot: GO\_CATALYTIC\_COMPLEX      
 Profile of the Running ES Score & Positions of GeneSet Members on the Rank Ordered List

  

| SYMBOL | TITLE | RANK IN GENE LIST | RANK METRIC SCORE | RUNNING ES | CORE ENRICHMENT || 1 | 2072 | ERCC4 | 7 | 1.130 | 0.0105 | Yes |
| 2 | 5514 | PPP1R10 | 11 | 1.087 | 0.0236 | Yes |
| 3 | 23248 | RPRD2 | 33 | 0.977 | 0.0209 | Yes |
| 4 | 9759 | HDAC4 | 46 | 0.925 | 0.0246 | Yes |
| 5 | 80746 | TSEN2 | 53 | 0.909 | 0.0328 | Yes |
| 6 | 57542 | KLHL42 | 59 | 0.876 | 0.0413 | Yes |
| 7 | 23338 | JADE2 | 71 | 0.855 | 0.0448 | Yes |
| 8 | 23054 | NCOA6 | 77 | 0.838 | 0.0527 | Yes |
| 9 | 3667 | IRS1 | 83 | 0.821 | 0.0604 | Yes |
| 10 | 55006 | TRMT61B | 101 | 0.782 | 0.0582 | Yes |
| 11 | 26272 | FBXO4 | 133 | 0.767 | 0.0447 | Yes |
| 12 | 2186 | BPTF | 159 | 0.717 | 0.0353 | Yes |
| 13 | 51434 | ANAPC7 | 166 | 0.710 | 0.0407 | Yes |
| 14 | 90025 | UBE3D | 167 | 0.710 | 0.0507 | Yes |
| 15 | 23590 | PDSS1 | 175 | 0.701 | 0.0552 | Yes |
| 16 | 57325 | CSRP2BP | 176 | 0.698 | 0.0651 | Yes |
| 17 | 23450 | SF3B3 | 178 | 0.697 | 0.0741 | Yes |
| 18 | 54815 | GATAD2A | 191 | 0.685 | 0.0744 | Yes |
| 19 | 85407 | NKD1 | 228 | 0.652 | 0.0555 | Yes |
| 20 | 55070 | DET1 | 234 | 0.648 | 0.0607 | Yes |
| 21 | 23001 | WDFY3 | 240 | 0.642 | 0.0659 | Yes |
| 22 | 6426 | SRSF1 | 243 | 0.637 | 0.0733 | Yes |
| 23 | 5701 | PSMC2 | 244 | 0.636 | 0.0823 | Yes |
| 24 | 1161 | ERCC8 | 255 | 0.625 | 0.0834 | Yes |
| 25 | 87178 | PNPT1 | 263 | 0.622 | 0.0867 | Yes |
| 26 | 152485 | ZNF827 | 268 | 0.618 | 0.0923 | Yes |
| 27 | 25852 | ARMC8 | 307 | 0.588 | 0.0709 | Yes |
| 28 | 26259 | FBXW8 | 318 | 0.583 | 0.0713 | Yes |
| 29 | 1021 | CDK6 | 324 | 0.579 | 0.0756 | Yes |
| 30 | 55697 | VAC14 | 329 | 0.576 | 0.0806 | Yes |
| 31 | 4297 | KMT2A | 337 | 0.573 | 0.0832 | Yes |
| 32 | 5356 | PLRG1 | 338 | 0.573 | 0.0913 | Yes |
| 33 | 2342 | FNTB | 344 | 0.570 | 0.0955 | Yes |
| 34 | 79184 | BRCC3 | 348 | 0.569 | 0.1012 | Yes |
| 35 | 55869 | HDAC8 | 352 | 0.566 | 0.1068 | Yes |
| 36 | 2965 | GTF2H1 | 356 | 0.563 | 0.1125 | Yes |
| 37 | 5718 | PSMD12 | 372 | 0.552 | 0.1085 | Yes |
| 38 | 1387 | CREBBP | 387 | 0.544 | 0.1053 | Yes |
| 39 | 54554 | WDR5B | 395 | 0.539 | 0.1074 | Yes |
| 40 | 79685 | SAP30L | 408 | 0.533 | 0.1055 | Yes |
| 41 | 54994 | GID8 | 415 | 0.526 | 0.1083 | Yes |
| 42 | 805 | CALM2 | 423 | 0.518 | 0.1101 | Yes |
| 43 | 801 | CALM1 | 424 | 0.518 | 0.1175 | Yes |
| 44 | 11177 | BAZ1A | 431 | 0.514 | 0.1200 | Yes |
| 45 | 54931 | TRMT10C | 446 | 0.507 | 0.1163 | Yes |
| 46 | 8607 | RUVBL1 | 451 | 0.506 | 0.1203 | Yes |
| 47 | 200933 | FBXO45 | 456 | 0.500 | 0.1242 | Yes |
| 48 | 27097 | TAF5L | 461 | 0.498 | 0.1281 | Yes |
| 49 | 10785 | WDR4 | 465 | 0.495 | 0.1328 | Yes |
| 50 | 10291 | SF3A1 | 466 | 0.493 | 0.1398 | Yes |
| 51 | 5295 | PIK3R1 | 467 | 0.493 | 0.1468 | Yes |
| 52 | 79960 | JADE1 | 468 | 0.493 | 0.1538 | Yes |
| 53 | 9690 | UBE3C | 470 | 0.493 | 0.1600 | Yes |
| 54 | 23468 | CBX5 | 484 | 0.485 | 0.1566 | Yes |
| 55 | 140775 | SMCR8 | 487 | 0.480 | 0.1619 | Yes |
| 56 | 8454 | CUL1 | 492 | 0.477 | 0.1655 | Yes |
| 57 | 8737 | RIPK1 | 493 | 0.477 | 0.1723 | Yes |
| 58 | 10940 | POP1 | 497 | 0.474 | 0.1766 | Yes |
| 59 | 11060 | WWP2 | 503 | 0.471 | 0.1794 | Yes |
| 60 | 1660 | DHX9 | 510 | 0.469 | 0.1813 | Yes |
| 61 | 617 | BCS1L | 513 | 0.467 | 0.1864 | Yes |
| 62 | 7334 | UBE2N | 546 | 0.449 | 0.1676 | No |
| 63 | 9416 | DDX23 | 553 | 0.445 | 0.1692 | No |
| 64 | 53615 | MBD3 | 561 | 0.441 | 0.1700 | No |
| 65 | 6632 | SNRPD1 | 563 | 0.440 | 0.1755 | No |
| 66 | 23404 | EXOSC2 | 609 | 0.418 | 0.1461 | No |
| 67 | 56339 | METTL3 | 613 | 0.417 | 0.1497 | No |
| 68 | 10445 | MCRS1 | 617 | 0.414 | 0.1532 | No |
| 69 | 22847 | ZNF507 | 618 | 0.413 | 0.1590 | No |
| 70 | 54512 | EXOSC4 | 623 | 0.411 | 0.1617 | No |
| 71 | 1108 | CHD4 | 627 | 0.408 | 0.1651 | No |
| 72 | 54663 | WDR74 | 628 | 0.408 | 0.1709 | No |
| 73 | 51728 | POLR3K | 646 | 0.401 | 0.1633 | No |
| 74 | 29890 | RBM15B | 651 | 0.400 | 0.1658 | No |
| 75 | 94005 | PIGS | 663 | 0.395 | 0.1628 | No |
| 76 | 79269 | DCAF10 | 668 | 0.394 | 0.1652 | No |
| 77 | 55958 | KLHL9 | 710 | 0.370 | 0.1383 | No |
| 78 | 178 | AGL | 711 | 0.368 | 0.1435 | No |
| 79 | 84172 | POLR1B | 718 | 0.365 | 0.1440 | No |
| 80 | 122809 | SOCS4 | 726 | 0.360 | 0.1436 | No |
| 81 | 899 | CCNF | 727 | 0.360 | 0.1487 | No |
| 82 | 8772 | FADD | 742 | 0.355 | 0.1428 | No |
| 83 | 64087 | MCCC2 | 747 | 0.352 | 0.1446 | No |
| 84 | 58509 | CACTIN | 748 | 0.351 | 0.1496 | No |
| 85 | 6605 | SMARCE1 | 749 | 0.349 | 0.1545 | No |
| 86 | 6241 | RRM2 | 755 | 0.346 | 0.1555 | No |
| 87 | 84148 | KAT8 | 757 | 0.344 | 0.1596 | No |
| 88 | 27316 | RBMX | 762 | 0.342 | 0.1613 | No |
| 89 | 8803 | SUCLA2 | 767 | 0.340 | 0.1630 | No |
| 90 | 10492 | SYNCRIP | 779 | 0.334 | 0.1591 | No |
| 91 | 80218 | NAA50 | 782 | 0.332 | 0.1622 | No |
| 92 | 4670 | HNRNPM | 790 | 0.330 | 0.1614 | No |
| 93 | 1736 | DKC1 | 791 | 0.330 | 0.1661 | No |
| 94 | 9221 | NOLC1 | 816 | 0.307 | 0.1516 | No |
| 95 | 9343 | EFTUD2 | 818 | 0.305 | 0.1552 | No |
| 96 | 11091 | WDR5 | 822 | 0.304 | 0.1571 | No |
| 97 | 26121 | PRPF31 | 825 | 0.301 | 0.1598 | No |
| 98 | 8835 | SOCS2 | 840 | 0.275 | 0.1527 | No |
| 99 | 10856 | RUVBL2 | 847 | 0.257 | 0.1517 | No |
| 100 | 8837 | CFLAR | 865 | -0.278 | 0.1423 | No |
| 101 | 51278 | IER5 | 874 | -0.283 | 0.1400 | No |
| 102 | 4008 | LMO7 | 909 | -0.335 | 0.1181 | No |
| 103 | 8881 | CDC16 | 963 | -0.383 | 0.0820 | No |
| 104 | 84919 | PPP1R15B | 979 | -0.394 | 0.0758 | No |
| 105 | 5163 | PDK1 | 982 | -0.394 | 0.0798 | No |
| 106 | 901 | CCNG2 | 998 | -0.405 | 0.0738 | No |
| 107 | 2146 | EZH2 | 1001 | -0.406 | 0.0780 | No |
| 108 | 51343 | FZR1 | 1005 | -0.407 | 0.0814 | No |
| 109 | 51646 | YPEL5 | 1014 | -0.413 | 0.0810 | No |
| 110 | 10616 | RBCK1 | 1042 | -0.438 | 0.0660 | No |
| 111 | 130617 | ZFAND2B | 1046 | -0.444 | 0.0700 | No |
| 112 | 26260 | FBXO25 | 1051 | -0.447 | 0.0732 | No |
| 113 | 5889 | RAD51C | 1059 | -0.451 | 0.0741 | No |
| 114 | 5111 | PCNA | 1077 | -0.460 | 0.0673 | No |
| 115 | 9567 | GTPBP1 | 1078 | -0.460 | 0.0738 | No |
| 116 | 196394 | AMN1 | 1079 | -0.461 | 0.0803 | No |
| 117 | 9939 | RBM8A | 1093 | -0.474 | 0.0768 | No |
| 118 | 5565 | PRKAB2 | 1094 | -0.474 | 0.0835 | No |
| 119 | 2729 | GCLC | 1123 | -0.497 | 0.0686 | No |
| 120 | 5277 | PIGA | 1126 | -0.500 | 0.0741 | No |
| 121 | 64795 | RMND5A | 1142 | -0.508 | 0.0696 | No |
| 122 | 1032 | CDKN2D | 1156 | -0.519 | 0.0668 | No |
| 123 | 146330 | FBXL16 | 1180 | -0.549 | 0.0565 | No |
| 124 | 8535 | CBX4 | 1184 | -0.555 | 0.0620 | No |
| 125 | 2152 | F3 | 1188 | -0.559 | 0.0676 | No |
| 126 | 57649 | PHF12 | 1212 | -0.588 | 0.0579 | No |
| 127 | 8408 | ULK1 | 1243 | -0.622 | 0.0432 | No |
| 128 | 114907 | FBXO32 | 1248 | -0.625 | 0.0489 | No |
| 129 | 8651 | SOCS1 | 1272 | -0.661 | 0.0402 | No |
| 130 | 7994 | KAT6A | 1279 | -0.666 | 0.0450 | No |
| 131 | 54800 | KLHL24 | 1287 | -0.677 | 0.0491 | No |
| 132 | 85417 | CCNB3 | 1299 | -0.700 | 0.0504 | No |
| 133 | 493861 | EID3 | 1315 | -0.729 | 0.0489 | No |
| 134 | 255488 | RNF144B | 1362 | -0.834 | 0.0247 | No |
| 135 | 4734 | NEDD4 | 1369 | -0.856 | 0.0321 | No |
Table: GSEA details [plain text format]

  

Fig 2: GO\_CATALYTIC\_COMPLEX      
 Blue-Pink O' Gram in the Space of the Analyzed GeneSet

  

Fig 3: GO\_CATALYTIC\_COMPLEX: Random ES distribution      
 Gene set null distribution of ES for **GO\_CATALYTIC\_COMPLEX**
